# Freestanding emergency department compliance with consumer protections: evidence from Texas

**DOI:** 10.1093/haschl/qxag095

**Published:** 2026-04-24

**Authors:** Daniel Marthey, Harish Sontam, Oscar Estrada-Gómez, Benjamin Ukert

**Affiliations:** Department of Health Policy and Management, Texas A&M University, College Station, TX 77843-1266, United States; Department of Health Policy and Management, Texas A&M University, College Station, TX 77843-1266, United States; Department of Health Policy and Management, Texas A&M University, College Station, TX 77843-1266, United States; Department of Health Policy and Management, Texas A&M University, College Station, TX 77843-1266, United States

**Keywords:** price transparency, freestanding emergency departments, consumer protections

## Abstract

**Introduction:**

Freestanding emergency departments (FrEDs) are a major source of emergency care in Texas, but pricing and network participation have historically lacked transparency. Texas House Bill (HB) 2041 aimed to strengthen consumer protections by requiring FrEDs to disclose facility status, standard fees, network participation, and prohibited deceptive advertising. We evaluated FrED compliance with HB 2041.

**Methods:**

We identified 16 reporting requirements spanning facility disclosure (*n* = 1), facility fees (*n* = 5), observation fees (*n* = 4), and insurance information (*n* = 6). Using a list of Texas FrEDs (*n* = 351) from the Department of State Health Services, we linked facility locations to hospital-based emergency department counts from the American Hospital Association Annual Survey and population data from the Census Bureau. We assessed compliance overall, by facility type (satellite vs independent FrED), reporting parameter, geography, and emergency department competition. Differences were evaluated using *t* tests.

**Results:**

FrED compliance with all 16 parameters ranged from 15% to 100%. Average compliance ranged from 64.5% and 44.7% among independent and satellite FrEDs, respectively. Among independent FrEDs, 11.5% complied with all requirements. The lowest compliance was observed for disclosure of facility and observation fees. Compliance was higher with greater emergency department competition.

**Conclusion:**

Findings suggest opportunities to enhance enforcement with Texas' consumer protections.

## Introduction

Freestanding emergency departments (FrEDs) have emerged as a rapidly growing model of health care delivery, representing 25% of all ED visits across approximately 350 facilities in Texas.^1,2^ To address longstanding concerns about surprise billing and opaque pricing, the Texas legislature passed House Bill (HB) 2041 in 2019,^3^ requiring FrEDs to disclose fees, network status, and other financial information in a standardized format on their public-facing websites. However, compliance with these rules remains unclear.

Freestanding EDs fall into 2 categories. Hospital-owned “satellite” facilities deliver emergency care at locations separate from the main campus but remain integrated with the parent hospital and are subject to Emergency Medical Treatment and Labor Act (EMTALA) requirements. Independently operated FrEDs are not hospital affiliated, not recognized by the Centers for Medicare and Medicaid Services (CMS) as certified providers, and cannot bill Medicare or Medicaid. In Texas, independent FrEDs are separately licensed by location while satellite FrEDs are exempt from licensing requirements.^4^

Price transparency is particularly important for FrEDs because their charges often exceed those of other similar care options. Evidence shows that there is little difference between commercial prices for the same services between FrEDs and hospital EDs,^5^ and FrED openings are associated with increases in spending on emergency care and medical debt.^6,7^ Ho et al^5^ found that the 20 most common FrED diagnoses closely mirrored those in urgent care centers but at 10 times the cost. In emergency situations, patients may not understand the services they are receiving or whether the provider is in-network,^8^ contributing to surprise medical bills.^9^

House Bill 2041 sought to improve transparency by requiring Texas FrEDs to post disclosures on facility status, fees, and insurance information.^3^ Whether FrEDs provide this information clearly and consistently remains uncertain. To assess compliance, we evaluated all 351 FrEDs operating in Texas in 2024 using 16 criteria derived from the law.

## Data and Methods

### Data

We obtained FrED data from the publicly available Texas Department of State Health Services website (downloaded November 11, 2024), which lists facility name, state license number, facility type, and address.^2^ Facilities classified as FrEDs with unique state license numbers were classified as independent while those sharing a license number with a hospital were classified as satellite FrEDs. (In Texas, hospital-owned FrEDs are exempt from licensing requirements and, instead, are listed in the facility data alongside the state license number of the parent hospital facility, while independently owned facilities are required to obtain a unique license number. The facility file, made available by the Texas Department of State Health Services, identifies the facility type of all health care facilities required to report data, including FrED facilities.) One facility that converted to an urgent care center was excluded, leaving 351 FrEDs (207 independent, 144 satellite), representing 156 (89 independent, 67 satellite) unique websites due to organizations operating multiple FrED locations. See Appendix Tables S1 and S2 for a list of all FrEDs included in our analysis. Using data from the American Hospital Association and US Census, we linked total population counts and FrEDs to hospital service areas (HSAs) and created a measure of competition defined as the number of hospital-based EDs in each HSA per 100 000 population. We then separated HSAs by quartile of competition level and compared compliance rates (lowest quartile [number of facilities = 47], medium [*n* = 189], high [*n* = 73], and very high [*n* = 42]). We also compared the characteristics (population size, median household income, percentage of the population with commercial insurance coverage) of FrED locations (cities) using data from the 2024 American Community Survey (ACS) 5-year estimates by place.

### Data collection

Between January 25 and 31, 2025, a primary reviewer identified each facility's website and recorded compliance with each parameter. A second reviewer conducted an independent review from May 27 to June 3, 2025. Discrepancies were reconciled by the research team. Each website was analyzed using a standardized set of 16 parameters based on the items mandated by HB 2041 (Table S3).

The parameters were organized into 4 categories—general facility identification, facility fee, observation fee, and insurance information—based on the requirements outlined in the bill text. The general facility identification category included 1 criterion: the website must explicitly state that the facility is a freestanding emergency medical care facility. The facility fee category included 5 criteria: (1) the website must explicitly state that this facility charges a facility fee for medical treatment, (2) mention that the facility charges rates comparable to a hospital emergency room, (3) include the facility's median facility fee, (4) include the range of possible facility fees, and (5) include the facility fees for each level of emergency care (eg, Current Procedural Terminology [CPT] codes 99281-99292) provided at the facility. The law also required the public posting of observation fees, another form of facility fee charged to patients who require prolonged observation or more intense treatment. The observation fee category included 4 criteria: the website must (1) explicitly state that this facility charges an observation fee for medical treatment and include (2) the facility's median observation fee, (3) a range of possible observation fees, and (4) the observation fees for each level of care provided at the facility. The insurance information category included 6 criteria: (1) the website must have a clear and explicit “insurance information” section; (2) mention that a facility or physician providing care may be an out-of-network provider for the patient's health benefit plan; (3) not claim to be an in-network provider unless officially a network provider for the health benefit plan; (4) avoid using phrases such as “takes” or “accepts” in reference to insurers, health maintenance organizations (HMOs), or health benefit plans unless in-network; (5) not display the name or logo of a health benefit plan issuer; and (6) mention that a physician providing medical care may bill separately from the facility.

### Statistical analysis

We created compliance rates for each facility and mapped the geographic distribution of FrEDs across Texas. Next, we compared the demographic composition (total population, median household income, percentage of the population with commercial insurance) of FrED city locations using 5-year estimates from the ACS. We then compared the overall compliance rate, the number of parameters met out of all 16 parameters, between independent and satellite FrEDs, using 2-sample *t* tests to assess whether their distributions differed. Freestanding ED status may be associated with compliance with specific provisions of HB 2041. For example, hospitals that are engaged in more frequent reporting may be more likely than independent FrEDs to have capacity to report average fees or health insurance network participation. To test this hypothesis, we examined compliance for each individual parameter by FrED status using *t* tests.

Compliance with HB 2041 could be also associated with organizational resources, regardless of FrED status, where smaller ownership organizations may have less capacity to address the new requirements. We did not observe direct measures of organization size, but we did observe whether FrEDs that operate multiple locations share the same website. To assess whether differences in compliance between satellite and independent FrEDs are associated with organizational resources we created a binary indicator (yes/no) to capture website sharing. We then examined average compliance between satellite and independent FrEDs, stratified by whether the FrED shared a website with multiple locations. Last, we evaluated whether compliance varied with ED competition, defined above.

## Results

Most independent and satellite FrEDs are clustered in major urban centers (Appendix Figure S1). Notably, address information suggests that 143 unique cities had at least 1 FrED and 43 cities house multiple FrEDs. Three cities—Houston, San Antonio, and Dallas—have a substantial number of independently owned FrEDs, each having at least 10 independently FrEDs, making up 19% of all independent FrEDs. Hospital FrEDs are also concentrated in major metropolitan areas, with Houston and San Antonio having more than 10 FrEDs, representing 20% of all satellite FrEDs. However, satellite FrEDs are also present in 37 cities without any independent FrED. Table S4 shows that, although satellite and independent FrEDs both locate in similarly sized urban centers, the city locations of independent FrEDs tend to have higher household incomes ($42 848 vs $39 224; *P* = .007) and larger commercially insured populations (66.7% vs 62.6%; *P* = .005) relative to satellite FrEDs. See Table S5 for the distribution of FrEDs in Texas by the top 16 cities.

**Figure 1:**
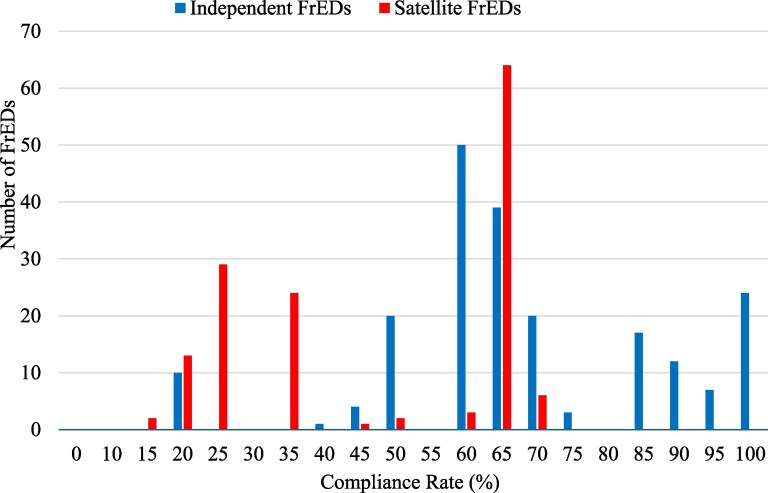
FrED compliance with HB 2041 consumer protections, by ownership type. Source: Authors' analysis of data collected from FrED facility websites. The sample included all 351 FrED facilities operating in Texas as of November 2024. Satellite FrEDs are owned by a hospital. Independent FrEDs are independently owned/operated. Abbreviations: FrED, freestanding emergency department; HB, House Bill.

The distribution of compliance rates for independent and satellite FrEDs is shown in Figure 1. The average compliance rate for independent FrEDs was 64.5% and was 44.7% for satellite FrEDs, suggesting that the majority of independent FrEDs are meeting many legal requirements for price transparency. The difference in compliance rates by facility type was statistically significant (*P* < .001). However, most are not complying across all parameters. Only 24 of the 207 independent FrEDs and 0 of the 144 satellite FrEDs complied with all 16 parameters. There were 16 independent FrEDs (7.7%) and 69 satellite FrEDs (47.9%) that had a compliance rate below 50%.

The average compliance rates by parameter are displayed in Table 1. The highest compliance rates for independent FrEDs were for “States that Facility is a FrED” and “Does NOT Claim to be In-Network unless officially a network provider for the benefit plan,” with compliance rates of 97.6% each. For satellite FrEDs, a 100% compliance rate was achieved for 1 parameter (“Does NOT Claim to be In-Network unless officially a network provider for the benefit plan”). Among independent FrEDs, the lowest compliance rate (21.7%) occurred for the parameter “States the Range of Observation Fees,” while 4 parameters (“States that the Facility Charges an Observation Fee,” “States the Range of Facility Fees,” “States the Median Observation Fee,” “States the Range of Observation Fees”) had a compliance rate of 0% for satellite FrEDs.

**Table 1:** FrED compliance with HB 2041 consumer protections, by parameter.

Parameter	Independent FrEDs, %	Satellite FrEDs, %	*P* value (*t* test)
States that Facility is a FrED	97.6	60.4	<.001
States that the Facility Charges a Facility Fee	95.2	52.8	<.001
States the Median Facility Fee	34.3	2.1	<.001
States the Range of Facility Fees	26.1	0.0	<.001
States the Facility Fee at Each Level	67.2	84.0	<.001
States that the Facility Charges an Observation Fee	24.6	0.0	<.001
States the Median Observation Fee	27.1	0.0	<.001
States the Range of Observation Fees	21.7	0.0	<.001
States the Observation Fee at Each Level	35.8	2.8	<.001
Clear and Explicit “Insurance Information” Section	84.5	70.1	.002
Mentions Comparable Charges to Hospital ER	94.7	52.8	<.001
Mentions Physician May Bill Separate from Facility	94.7	52.8	<.001
Mentions Out-of-Network Provider for Benefit Plan	93.7	52.8	<.001
Does NOT Claim to be In-Network unless officially a network provider for the benefit plan	97.6	100.0	.025
Does NOT use “takes” or “accepts” for insurers, HMOs, or benefit plans unless in-network	77.3	89.6	.002
Does NOT display name or logo of benefit plan if out-of-network	89.86	98.61	<.001

Abbreviations: ER, emergency room; FrED, freestanding emergency department; HB, House Bill; HMO, health maintenance organization.

Source: Authors' analysis of data collected from FrED facility websites. The sample included all 351 FrED facilities operating in Texas as of November 2024. Satellite FrEDs are owned by a hospital. Independent FrEDs are independently owned/operated.

Differences by parameter emerged by independent and satellite FrEDs in terms of stating that the facility is an FrED (97.6% vs 60.4%) and that the provider charges a facility fee (95.2% vs 52.8%). The contrast in compliance rates for identifying the provider and the facility fee information can be partially the result of independent FrEDs using a common template to provide information under the “Insurance” section of their websites, regardless of ownership of the independent FrED, potentially because they relied on standard legal or administrative language especially prepared for independent FrEDs. However, this did not lead the facility to have their own website. For the 207 independent FrEDs, there were only 89 unique websites, as some of the FrEDs were owned by the same parent company and had the same website. This was also seen with satellite FrEDs, as there were 144 FrEDs and only 67 unique websites. Generally, each location had its own page under the same website, with the same layout of information.

In Table S6 we show that satellite facilities are, regardless of sharing a website, on average less compliant compared with independent FrEDs. For example, the compliance rate for facilities not sharing a website was 42.9% for satellites and 65.3% for independent FrEDs (*P* < .001). For those sharing a website the compliance rate was 45.2% for satellite and 66.3% for independent FrEDs (*P* < .001). This suggests that hospitals that may use a decentralized approach, typically a feature of not having a large parent organization, may be associated with marginally lower compliance rates, possibly due to lower centralized oversight and less-standardized website templates.

House Bill 2041 states that FrEDs must have an explicit “Insurance” section, but many FrEDs commonly have an “Insurance and Transparency” section. To maintain consistency, these FrEDs were still noted as compliant in the data because they included “Insurance.” However, there were a few FrEDs that named the section as only “Price Transparency,” and these FrEDs were noted as noncompliant. Freestanding EDs generally included the information in the “Frequently Asked Questions” and “Billing” sections, which could potentially be a way of hiding the notices; however, we still considered those to be compliant if information was provided. The parameter “Clear and Explicit ‘Insurance Information’ Section” had a high compliance rate for independent and satellite FrEDs (84.5% and 70.1%, respectively). Most FrEDs complied (77.3% and 89.6%, respectively) with the prohibition of misleading words (eg, “takes,” “accepts”) regarding network participation. However, we observed similar language including the terms “welcome,” “work with,” and “recognize.”

Freestanding ED compliance increased with HSA-level competition (Table 2). The FrEDs in HSAs with the least competition by hospital-based EDs (≤0.23 hospital-based EDs per 100 000 population) had a compliance rate of 48%, 58% in medium competitive HSAs (0.23 to 0.54 hospital-based EDs per 100 000 population), 58% in highly competitive HSAs (0.54 to 1.28 hospital-based EDs per 100 000 population), and 61% in the highest competition HSAs (>1.28 hospital-based EDs per 100 000 population). Differences between independent and satellite facilities emerged, with independent FrEDs having much higher compliance rates, more than 65%, across the competitive landscape. Among satellite FrEDs we observed a clear increase in compliance rates by competitive quartile, with the compliance rate increasing from 340% in the least competitive HSA to 45% in the most competitive HSA, suggesting that the availability of substitute emergency care facilities may play a role in how satellite FrEDs operate.

**Table 2:** Average FrED compliance with HB 2041, by emergency department market competition.

HSA competition	Overall compliance rate, %	Compliance rate of independent FrEDs, %	Compliance rate of hospital FrEDs, %	*P* value (*t* test)
Low (*n* = 47)	54	68	40	<.001
Medium (*n* = 189)	58	65	46	<.001
High (*n* = 73)	58	66	45	<.001
Very high (*n* = 42)	61	68	45	.002

Abbreviations: FrED, freestanding emergency department; HB, House Bill; HSA, hospital service area.

Source: Authors' analysis of data collected from FrED facility websites. The sample included all 351 FrED facilities operating in Texas as of November 2024. Satellite FrEDs are owned by a hospital. Independent FrEDs are independently owned/operated. Facility-level compliance rates are calculated by the level of competition measured with the number of hospital-based emergency departments per 100 000 population in the HSA. Emergency room counts were obtained from the American Hospital Association (AHA) Annual Survey.

## Discussion

Transparency in pricing and network participation is important for helping patients anticipate out-of-pocket costs. House Bill 2041 was designed to improve the transparency of emergency care, yet compliance in 2025 remained limited, with 64.5% and 44.7% among independent and satellite FrEDs, respectively. Only 11.5% of independent and no hospital satellite FrEDs were in full compliance with HB 2041.

Even where compliance was relatively high, the placement of information often undermined the purpose of the rule. For example, most FrEDs disclosed their status as a FrED only in pricing and insurance subsections of their websites. Even among the websites that mentioned “FrED” on their home pages, the term was often used minimally, usually appearing only once. Instead, these facilities preferred to market themselves as “24/7 care facilities,” “emergency centers,” or “urgent care facilities.” Additionally, regarding network participation, many FrEDs used terms such as “welcome,” “work with,” and “recognize.” This choice of language is important because it directly influences patient perceptions about services and potential out-of-pocket expenses. If a patient in need of emergency care sees a facility labeled as an “emergency center” rather than explicitly as an FrED, they may incorrectly assume that it is part of a hospital system or that it follows the same regulations. This misinterpretation could lead patients to seek care at FrEDs without realizing the potential financial implications, as FrEDs remain a relatively new concept.

Given the somewhat different billing practices and service structures of FrEDs compared with urgent care centers, clarity on pricing is important even if patients are protected against surprise bills through the federal No Surprises Act.^10^ A visit to an FrED will still trigger copays and co-insurance rates based on health insurance benefit coverage for ED care, which can lead to hundreds or thousands of dollars of out-of-pocket spending. Further, while most EDs, including hospital satellite FrEDs, are recognized by the Centers for Medicare and Medicaid Services (CMS), independent FrEDs are not, preventing them from billing Medicare or Medicaid. This distinction substantially affects the cost of care for the individuals with low income and elderly individuals. The failure to clearly identify charges occurring under emergency care may mislead patients into assuming these facilities operate under the same service and billing models.

Although HB 2041 sought to protect consumers, patients who are non–English-speaking or those who have other disabilities preventing them from accessing information online or in paper format may not benefit from the enhanced regulations. To our knowledge, facilities are only required to make paper copy disclosures available to patients in English or Spanish, while other posted notices online and within clinic settings have no such requirements. Additionally, patients who arrive at an FrED in need of immediate medical intervention and those without adequate supportive services may not learn about a facility's FrED status until after they receive treatment, undermining consumer protections.

Our findings are consistent with work evaluating the federal hospital price transparency law.^11^ Effective in 2021, the CMS price transparency rule required hospitals to post a comprehensive set of standard charges on their websites in machine-readable (eg, CSV, XML) format and a limited set of 300 “shoppable” services in a consumer-friendly format. However, early evidence has suggested that most hospitals are not compliant.^12-14^ One recent study examined hospital compliance with the price transparency rule using a nationally representative random sample of 470 general acute hospitals. They found that, by 2022, 46% were in compliance with both formats and 47% with the machine-readable format.^12^ These estimates are qualitatively aligned with the 45% compliance we observed among hospital-owned satellite FrEDs.

Another study found that some hospitals were hiding certain service prices or providing prices for services they do not treat, and only 6% of the sampled hospitals posted completely accurate prices and services.^14^ Hospital compliance with CMS price transparency rules could directly impact satellite FrED compliance with HB 2041, particularly if the FrED and parent hospital share the same website.

Policymakers and regulators should consider stronger enforcement mechanisms to ensure FrED compliance with transparency requirements. This could include more frequent audits of FrED websites and a more robust system for tracking and penalizing noncompliant facilities, such as escalating fines for repeated violations. Previous evidence has shown that increased penalties led to substantial increases in compliance with hospital price transparency rules.^15^ Under current Texas law the penalty is up to $1000 per violation where each day “may be considered” a distinct violation.

### Limitations

The main limitation of our study was the inability to evaluate compliance by providers over time as our data were collected within the same half-year. Nevertheless, the findings provide timely evidence on a law that, to our knowledge, has not been previously assessed. Second, as one of the first states to enact price transparency rules for FrEDs, Texas is an important case to study, but our results might not be generalizable to other states.

## Conclusion

Overall, FrED compliance with Texas HB 2041 remains limited and varies by facility type. Improving transparency is critical to protecting consumers from unexpected medical costs and ensuring meaningful financial decision-making.

## Supplementary Material

qxag095_Supplementary_Data

## References

[1] Marthey D, Ramy M, Ukert B. Who do freestanding emergency departments treat? Comparing Texas hospitals to satellite and independent freestanding departments in 2021 and 2022. Health Serv Res. 2024;59(4):e14304. 10.1111/1475-6773.14304 38515240 PMC11249826

[2] Texas Department of State Health Services (DSHS) . Facility reporting requirements. 2025. Accessed November 11, 2024. https://www.dshs.texas.gov/center-health-statistics/texas-health-care-information-collection/facility-reporting-requirements

[3] Texas Legislature . House Bill 2041. 86th Legislature, Regular Session. 2019. Accessed November 6, 2025. https://capitol.texas.gov/tlodocs/86R/billtext/html/HB02041F.htm

[4] Texas Administrative Code . Title 26, Part 1, Chapter 509. 2024. Accessed November 6, 2025. https://texas-sos.appianportalsgov.com/rules-and-meetings?chapter=509&interface=VIEW_TAC&part=1&subchapter=B&title=26

[5] Ho V, Metcalfe L, Dark C, et al Comparing utilization and costs of care in freestanding emergency departments, hospital emergency departments, and urgent care centers. Ann Emerg Med. 2017;70(6):846–857, e3. 10.1016/j.annemergmed.2016.12.006 28262320

[6] Ho V, Xu Y, Akhter M. Freestanding emergency department entry and market-level spending on emergency care. Acad Emerg Med. 2019;26(11):1221–1231. 10.1111/acem.13848 31637823 PMC6899627

[7] Marthey D, Ukert B, Andreyeva E. Medical debt and entry of satellite freestanding emergency departments. JAMA Netw Open. 2025;8(7):e2522876. 10.1001/jamanetworkopen.2025.22876 40699571 PMC12287832

[8] Giannouchos TV, Ukert B, Wright B. Concordance in medical urgency classification of discharge diagnoses and reasons for visit. JAMA Netw Open. 2024;7(1):e2350522. 10.1001/jamanetworkopen.2023.50522 38198140 PMC10782231

[9] Cooper Z, Scott Morton F, Shekita N. Surprise! Out-of-network billing for emergency care in the United States. J Polit Econ. 2020;128(9):3626–3677. 10.1086/708819

[10] Code of Federal Regulations . No Surprises Act. 2021. Accessed November 6, 2025. https://www.ecfr.gov/current/title-45/subtitle-A/subchapter-B/part-149

[11] Centers for Medicare & Medicaid Services (CMS) . Hospital price transparency. 2025. Accessed November 6, 2025. https://www.cms.gov/priorities/key-initiatives/hospital-price-transparency

[12] Nikpay S, Carroll C, Golberstein E, Abraham JM. Playing by the rules? Tracking U.S. hospitals' responses to federal price transparency regulation. J Healthc Manag. 2024;69(1):45–58. 10.1097/JHM-D-23-00014 38175534

[13] Henderson MA, Mouslim MC. Low compliance from big hospitals on CMS's hospital price transparency rule. Health Aff Forefr. 2021. 10.1377/forefront.20210311.899634

[14] Mead M, Ibrahim AM. Over- and underreporting of prices: most hospitals are not compliant with the hospital price transparency rule. Health Aff Sch. 2024;2(9):qxae099. 10.1093/haschl/qxae099 39220579 PMC11363865

[15] Kong E, Ji Y. Provision of hospital price information after increases in financial penalties for failure to comply with a US federal hospital price transparency rule. JAMA Netw Open. 2023;6(6):e2320694. 10.1001/jamanetworkopen.2023.20694 37378982 PMC10308252

