# Supplementary material for: Freestanding emergency department compliance with consumer protections: evidence from Texas
Source: Health Aff Sch. 2026 Apr 24;4(5):qxag095. doi: 10.1093/haschl/qxag095 (PMC13186269; doi:10.1093/haschl/qxag095)
Supplement: qxag095_Supplementary_Data [file qxag095_supplementary_data.zip › qxag095_Supplementary_Data.docx]

**Freestanding Emergency Department Adherence with Consumer Protection Requirements: Evidence from Texas**

Supplemental Appendix

**Figure S1**. Distribution of FrEDs in Texas, 2024

**
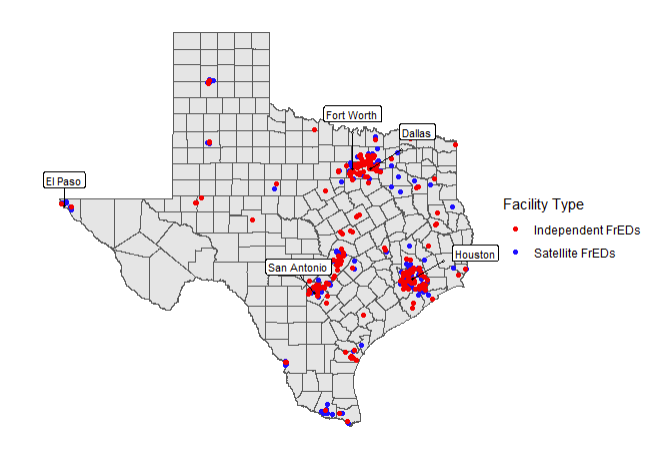
**

Source: List of facilities required to report emergency department data obtained from the Texas Department of State Health Services (DSHS), Texas Health Care Information Collection (THCIC). Notes: The data were obtained on November 11, 2024. Satellite FrEDs are owned by a hospital. Independent FrEDs are independently owned/operated.

**Table S1.** List of Satellite FrEDs Operating in Texas as of November 2024

| **FrED Name** | **Address** | **City** |
| --- | --- | --- |
| Hunt Regional Emergency Medical Center of Commerce | 2800 Highway 24 | Commerce |
| Hunt Regional Emergency Medical Center at Quinlan | 738 E Quinlan Pkwy | Quinlan |
| Paris Regional Emergency Center | 3055 NE Loop 286 | Paris |
| Baylor St Lukes Emergency Center Holcombe | 2727 West Holcombe Blvd | Houston |
| Houston Methodist Emergency Care Center at Voss | 1635 South Voss Road | Houston |
| The Methodist Hospital Emergency Care Center | 2615 Southwest Freeway | Houston |
| Pearland Emergency Care Center | 11525 Broadway | Pearland |
| Houston Methodist Emergency Care Center in Sienna Plantation | 8200 Hwy 6 | Missouri City |
| Houston Methodist Willowbrook Hospital ER | 27560 US 290 Frontage Rd | Cypress |
| Houston Methodist Emergency Care Center Magnolia | 18230 FM 1488 Rd | Magnolia |
| Houston Methodist Emergency Care Center in Spring | 5303 FM 2920 | Spring |
| Houston Methodist Emergency Care Center in Cinco Ranch | 26000 FM 1093 | Katy |
| Houston Methodist Emergency Care Center League City | 1310 E League City Pkwy | League City |
| Houston Methodist Emergency Care Center at the Woodlands | 3759 FM 1488 Rd | The Woodlands |
| HM Deer Park ECC | 3701 Center St | Deer Park |
| South Texas Health System ER Alamo | 140 E Frontage Road | Alamo |
| South Texas Health System ER McColl | 4702 South McColl Road | Edinburg |
| South Texas Health System ER Monte Cristo | 3615 N Interstate 69C | Edinburg |
| South Texas Health System ER Ware Road | 3700 W Nolana Ave | McAllen |
| South Texas Health System ER Mission | 900 E Expressway 83 | Mission |
| South Texas Health System ER Pharr | 601 W Ridge Rd | Pharr |
| South Texas Health System ER Weslaco | 330 W Expressway 83 | Weslaco |
| Methodist ER Boerne | 134 Menger Springs | Boerne |
| Methodist ER Helotes | 12285 Bandera Rd | Helotes |
| Methodist ER New Braunfels | 1850 W TX-46 #109 | New Braunfels |
| Methodist ER Alamo Heights | 250 E Basse Rd | San Antonio |
| Methodist ER City Base | 3154 SE Military Drive #103 | San Antonio |
| Methodist ER Converse | 6402 Mallard Meadow | San Antonio |
| Methodist ER De Zavala | 12805 W I-10 | San Antonio |
| Methodist ER Legacy Trails | 9211 Potranco Rd | San Antonio |
| Methodist ER Nacogdoches | 13434 Nacogdoches Rd | San Antonio |
| Methodist Westover Hills Emergency Center | 5538 West Loop 1604 North | San Antonio |
| Methodist ER Bulverde | 20475 TX-46 | Spring Branch |
| Memorial Hermann Greater Heights Convenient Care Center | 1431 Studemont St | Houston |
| Memorial Hermann Convenient Care Center - League City | 2555 S Gulf Freeway | League City |
| Memorial Hermann Convenient Care Center - Spring | 7474 N Grand Parkway | Spring |
| Memorial Hermann 24 HR Emergency Care - The Woodlands | 9950 Woodlands Parkway | The Woodlands |
| Memorial Hermann South Katy Convenient Care Center | 22430 Grand Corner Dr | Katy |
| Memorial Hermann Covenient Care Center Summer Creek | 14201 E Sam Houston Parkway North | Houston |
| Memorial Hermann Convenient Care Center Kingwood | 4533 Kingwood Drive | Kingwood |
| Memorial Hermann Convenient Care Center - Sienna Plantation | 8780 Highway 6 | Missouri City |
| Las Palmas Del Sol Emergency Center - West | 1535 North Resler | El Paso |
| Las Palmas Del Sol Emergency Center - Zaragoza | 1951 Zaragoza Road | El Paso |
| Las Palmas Del Sol Healthcare - Horizon | 13401 Gateway Blvd West | El Paso |
| Las Palmas Del Sol Healthcare - Northeast | 4740 Loma del Sur | El Paso |
| Laredo Medical Center North Central ER | 9811 McPherson Road | Laredo |
| HCA Houston ER 24/7 - Alvin | 2860 S Gordon | Alvin |
| HCA Houston ER 24/7 - Friendswood | 225 E. Parkwood Ave | Friendswood |
| HCA Houston ER 24/7 - Pearland | 2906 Broadway | Pearland |
| HCA Houston ER 24/7 - Texas City | 3302 Palmer Highway | Texas City |
| HCA Houston ER 24/7 - Mission Bend | 8910 Highway 6 S | Houston |
| HCA Houston ER 24/7 - Westchase | 11103 Westheimer Rd | Houston |
| HCA Houston ER 24/7 - Stafford | 3531 South Main St | Stafford |
| HCA Houston ER 24/7 - North Channel | 6191 East Sam Houston Pkwy | Houston |
| HCA Houston ER 24/7 - Fairmont | 6002 Fairmont Parkway | Pasadena |
| HCA Houston ER 24/7 - Cleveland | 1103 East Houston Street | Cleveland |
| HCA Houston ER 24/7 - Fry Road | 26271 Northwest Freeway | Cypress |
| HCA Houston ER 24/7 - Towne Lake | 9645 Barker Cypress Road | Cypress |
| HCA Houston ER 24/7 - Copperfield | 5835 Hwy 6 North | Houston |
| HCA Houston ER 24/7 - Willowbrook | 22475 Tomball Parkway | Houston |
| HCA Houston ER 24/7 - Atascocita | 5324 Atascocita Rd | Humble |
| HCA Houston ER 24/7 - Fall Creek | 9711 N Sam Houston Pkwy East | Humble |
| HCA Houston ER 24/7 - Northwest | 13338 Tomball Parkway | Houston |
| HCA Houston ER 24/7 - Spring Rayford | 621 Rayford Rd | Spring |
| HCA Houston ER 24/7, A Department of HCA Houston Healthcare Pearland | 14476 Hillcroft Ave | Houston |
| HCA Houston ER 24/7 - Cypress | 5003 Cypress Creek Pkwy | Houston |
| HCA Houston ER 24/7 - Bellaire | 5413 South Rice Ave | Houston |
| HCA Houston ER 24/7 - Memorial City | 1014 Wirt Road | Houston |
| HCA Houston ER 24/7 - Montrose | 3209 Montrose Blvd | Houston |
| HCA Houston ER 24/7 - TC Jester | 1925 East TX Jester | Houston |
| Texas Health Burleson | 2750 Southwest Wilshire Blvd | Burleson |
| Texas Health Willow Park | 101 Crowne Pointe Blvd | Willow Park |
| Texas Health Prosper | 1970 W University Drive | Prosper |
| UMC East Emergency Department | 1521 Joe Battle | El Paso |
| UMC Northeast Emergency Department | 4669 Cohen Avenue | El Paso |
| CHRISTUS Mother Frances Emergency Center - Athens | 1509 S Palestine St | Athens |
| Christus Mother Frances Emergency Center - Canton | 18780 I-20 | Canton |
| CHRISTUS Mother Frances Emergency Center - Lindale | 3203 S Main St | Lindale |
| Children's Hospital of San Antonio Emergency Center - Stone Oak | 1434 E Sonterra Blvd | San Antonio |
| Children's Hospital of San Antonio Emergency Center - Westover Hills | 11130 Christus Hills | San Antonio |
| Christus Santa Rosa Emergency Center - Creekside | 244 Creekside Crossing | New Braunfels |
| Christus Santa Rosa Emergency Center - Alon | 11503 NW Military Highway | San Antonio |
| Christus St Michael Emergency Center | 4250 Gibson Lane | Texarkana |
| Christus Good Shepherd Emergency Department Kilgore | 1612 South Henderson Blvd | Kilgore |
| Doctors Hospital Emergency Room Saunders | 1300 E Saunders St | Laredo |
| Doctors Hospital Emergency Room South | 2901 Jaime Zapata Hwy | Laredo |
| Doctors Hospital Emergency Room Wright Ranch | 2801 Cuatro Vien | Laredo |
| Northwest Emergency at Eastern | 4409 E Interstate 40 | Amarillo |
| Northwest Emergency at Tascosa | 4207 W Amarillo Blvd | Amarillo |
| Northwest Emergency at Town Square | 8960 Hillside Road | Amarillo |
| Northwest Emergency on Georgia | 4121 S Georgia Street | Amarillo |
| Shelby County Emergency Services | 5100 Loop 500 East | Center |
| Lake Granbury Emergency Center | 5309 E Hwy 377 | Granbury |
| Hendrick ED South | 5310 Buffalo Gap Rd | Abilene |
| Longview Regional Emergency Center | 120 E Loop 281 | Longview |
| Rio Grande Regional Hospital 24 Hour Emergency Care - Edinburg | 2744 W University Drive | Edinburg |
| Rio Grande Regional Hospital 24 Hour Emergency Care - McAllen/Mission | 5100 West Expressway 83 | McAllen |
| Rio Grande Regional Hospital 24 Hour Emergency Care - San Juan | 200 East Expressway 83 | San Juan |
| ER 24/7 Mission | 102 W Griffin Parkway | Mission |
| Northshore Emergency Center | 1702 Highway 181 North | Portland |
| ER 24/7 Rockport | 400 Enterprise Blvd | Rockport |
| St. David's Emergency Center - Bastrop | 3201 Highway 71 East | Bastrop |
| St. David's Emergency Center - Bee Cave | 12813 Gallerina Circle | Bee Cave |
| St. David's Emergency Center - Leander | 4181 US 183 | Leander |
| St Davids North Austin Medical Center Emergency Department | 18917 Limestone Commercial Dr | Pflugerville |
| CHI St Lukes Health Emergency Center - Pasadena | 5161 E Sam Houston Pkwy | Pasadena |
| CHI St Lukes Health Emergency Center - Huntsville | 540 I 45 South | Huntsville |
| ER at Anna | 2710 Hackberry Drive | Anna |
| ER at Sherman | 4226 US Highway 75 North | Sherman |
| Cedar Park Regional Emergency Center - Leander | 1751 Crystal Falls Parkway | Leander |
| Baylor Scott & White Emergency Center - Forney | 757 E US Hwy 80 | Forney |
| Baylor Scott & White Emergency Center - Wylie | 2300 FM 544 | Wylie |
| Valley Baptist Emergency Center - Harlingen | 1725 N Ed Carey Dr | Harlingen |
| Valley Baptist Emergency Center - Brownsville | 2073 E Rubin M Torres Sr Blvd | Brownsville |
| Baptist Emergency Hospital-Shavano Park | 4103 North Loop 1604 West | San Antonio |
| Baptist Neighborhood Hospital Converse | 6491 Woodlake Parkway | San Antonio |
| Altus Baytown - Baker | 1404 W Baker Rd | Baytown |
| Altus Baytown ER - Garth | 6051 Garth Road | Baytown |
| Altus Baytown ER Kingwood | 1120 Kingwood Drive | Kingwood |
| Altus Baytown ER - Broadway | 11130 W BROADWAY STREET | PEARLAND |
| Medical City ER White Settlement | 9650 White Settlement Rd | Fort Worth |
| Medical City ER Garland | 3318 West Buckingham Road | Garland |
| Medical City ER Grand Prairie | 5203 Lake Ridge Parkway | Grand Prairie |
| Medical City ER Red Oak | 401 East Ovilla Road | Red Oak |
| Medical City Argyle | 7214 Crawford Rd | Argyle |
| Medical City ER Stonebridge | 8995 University Drive | McKinney |
| Medical City ER Haslet | 13172 NW Hwy 287 | Fort Worth |
| Medical City ER Saginaw | 766 West Bailey Boswell Road | Saginaw |
| Cleveland Emergency Hospital - Porter | 24540 FM 1314 Road | Porter |
| Cleveland Emergency Department - Woodlands | 26226 I-45 North | Spring |
| The Hospitals of Providence Emergency Room Edgemere | 12101 Edgemere Blvd | El Paso |
| The Hospitals of Providence Emergency Room Montwood | 1890 George Dieter Drive | El Paso |
| West Plano Emergency Room | 2000 Dallas Parkway | Plano |
| WYLIE ER | 508 S HIGHWAY 78 | WYLIE |
| CapRock 24 Hour Emergency | 948 William D Fitch | College Station |
| UT Health Cedar Creek Lake Emergency Center | 100 Municipal Drive | Gun Barrel City |
| UT Health South Broadway Emergency Center | 6210 S Broadway Ave | Tyler |
| Covenant Health Emergency Center - Milwaukee Ave | 7905 Milwaukee Ave | Lubbock |
| Covenant Health Emergency Center - Quaker Ave | 10205 Quaker Ave | Lubbock |
| STAT Emergency Center | 1023 Bob Bullock Loop | Laredo |
| Cedar Park Emergency Center LLC | 1464 E Whitestone Blvd | Cedar Park |
| Exceptional Emergency Center Beaumont a dept of Altus Lumberton Hospital | 4755 Eastex Fwy | Beaumont |
| Exceptional Emergency Center Orange a dept of Altus Lumberton Hospital | 1321 N 16th St | Orange |
| Exceptional Emergency Center Port Arthur a dept of Altus Lumberton Hospital | 3330 E Farm to Market Road 365 | Port Arthur |

**Table S2.** List of Independent FrEDs Operating in Texas as of November 2024

| **FrED Name** | **Address** | **City** |
| --- | --- | --- |
| Advance Er | 12338 Inwood Rd | Dallas |
| Advance Er | 5201 Lovers Lane | Dallas |
| Ally Medical Emergency Room - Bastrop | 512 Highway 71 West | Bastrop |
| Ally Medical Emergency Room - Buda | 15610 Ih 35 | Buda |
| Ally Medical Emergency Room - Central Austin | 5525 Burnet Rd Suite A | Austin |
| Ally Medical Emergency Room - Clear Lake | 3351 Clear Lake Blvd Suite 100 | Houston |
| Ally Medical Emergency Room - Dripping Springs | 333 E Hwy 290 #350 | Dripping Springs |
| Ally Medical Emergency Room - Round Rock | 2105 E Palm Valley Blvd | Round Rock |
| Ally Medical Emergency Room - South Austin | 8721 Manchaca Road | Austin |
| Ally Medical Emergency Room - Spring | 2490 Fm 2920 Suite 100 | Spring |
| Altus Lake Jackson Lp | 200 Oak Drive South | Lake Jackson |
| Altus Waxahachie Lp | 1791 N Hwy 77 | Waxahachie |
| Amarillo South Texas Emergency Center Llc | 5800 S Coulter St | Amarillo |
| Amarillo West Texas Emergency Center Llc | 2105 S Western St | Amarillo |
| Tyler Texas Emergency Center Llc | 2222 E Southeast Loop 323 | Tyler |
| Brownsville Texas Emergency Center Llc | 449 East Alton Gloor Blvd | Brownsville |
| Eastchase Texas Emergency Center Llc | 1251 East Chase Parkway | Fort Worth |
| Harlingen Texas Emergency Center Llc | 6902 West Expressway 83 | Harlingen |
| Livingston Texas Emergency Center Llc | 111 Emergency Road | Livingston |
| Americas Er | 13902 Spring Cypress Rd Suite A | Cypress |
| Americas Er | 32784 Fm 2978 Suite A | Magnolia |
| Angleton Er Pllc | 1116 E Mulberry St | Angleton |
| Ascent Emergency Medical Center | 2280 Holcombe Blvd | Houston |
| Austin Emergency Center | 4015 South Lamar Blvd | Austin |
| Austin Emergency Center | 1801 E 51St St Bldg H | Austin |
| Austin Emergency Center | 10407 Jollyville Road | Austin |
| Austin Emergency Center | 13435 N Hwy 183 Ste 311 | Austin |
| Austin Emergency Center | 2020 E Riverside Drive | Austin |
| Austin Emergency Center | 15100 Fm 1825 | Pflugerville |
| Bellaire Er | 5302 Bellaire Boulevard | Bellaire |
| Care Plus Emergency Room | 8111 West Grand Parkway South | Richmond |
| Castle Hills Er | 4228 N Josey Ln | Carrollton |
| Clear Choice Er Llc | 7105 N Bartlett Ave Suite #101 | Laredo |
| Village Emergency Room Llc | 17030 Nw Freeway Building A | Jersey Village |
| Clear Creek Emergency Room Llc | 3725 E League City Parkway Ste 150 | League City |
| Community First Er | 1101 East Blvd | Deer Park |
| Complete Care Camp Bowie | 6006 Camp Bowie | Fort Worth |
| Complete Emergency Care City Base | 2619 Se Military Dr Suite 101 | San Antonio |
| Complete Emergency Care De Zavala Llc | 4999 De Zavala Road | San Antonio |
| Complete Emergency Care I Llc | 10628 Culebra Road Suite 200 | San Antonio |
| Complete Emergency Care La Vernia Llc | 102 S Fm 1346 Suite 2 | La Vernia |
| Complete Emergency Care Southlake | 321 W Southlake Blvd Suite 140 E | Southlake |
| Fossil Creek Complete Care | 22250 Bulverde Road Suite 120 | San Antonio |
| Lakeway Complete Care Llc | 1518 Ranch Road 620 South Suite 200 | Lakeway |
| Tlc Complete Care | 7330 South Staples Street | Corpus Christi |
| Tyler Complete Care | 1809 Capital Dr | Tyler |
| Westlake Complete Care Llc | 6836 Bee Caves Rd Suite 112 | Austin |
| Concho Valley Er | 5709 Sherwood Way | San Angelo |
| Coppell Er | 720 N Denton Tap Road | Coppell |
| El Paso Emergency Room | 3281 Joe Battle Blvd | El Paso |
| El Paso West Emergency Room | 351 Redd Rd | El Paso |
| Emergency Care Of Floresville | 101 Wilson Dr Ste 102 | Floresville |
| Er Now | 4121 Southwest Pkwy | Wichita Falls |
| Er Of Dallas | 4535 Frankford Road | Dallas |
| Er Of Fort Worth | 4561 Heritage Trace Parkway | Fort Worth |
| Er Of Mesquite | 1745 N Belt Line Rd | Mesquite |
| Er Of Texas - Highland Village | 3160 Justin Road | Highland Village |
| Er Of Texas - Hillcrest | 6215 Hillcrest Ave | Dallas |
| Er Of Texas Colleyville | 5000 Highway 121 | Colleyville |
| Er Of Texas Frisco | 16300 State Highway 121 | Frisco |
| Er Of Texas Hurst | 824 Airport Frwy | Hurst |
| Er Of Texas Little Elm | 2800 Little Elm Parkway | Little Elm |
| Er Of Texas Texoma | 115 W Travis St | Sherman |
| Er Of Watauga | 5401 Basswood Blvd | Fort Worth |
| Er On Soncy | 3530 S Soncy Rd | Amarillo |
| Excel Er Nacogdoches | 1420 North St | Nacogdoches |
| Excel Er Odessa | 6131 E Hwy 191 | Odessa |
| Express Er | 1551 W Central Avenue | Temple |
| Express Er | 1411 N Valley Mills Drive | Waco |
| Express Er | 4157 Buffalo Gap Rd | Abilene |
| Express Er | 980 Knights Way Bld 1 | Harker Heights |
| Fair Oaks Emergency Room | 27638 Ih-10 West | Boerne |
| Fairfield Emergency Room | 15103 Mason Rd Ste E1 | Cypress |
| Family First Er | 19143 W Lake Houston Pkwy | Humble |
| Family First Er Baytown | 5410 East Freeway | Baytown |
| Frisco Er | 12600 Rolater Rd | Frisco |
| Frontline Er (Dallas) | 7331 Gaston Road Suite 180 | Dallas |
| Frontline Er (Richmond) | 7051 Fm 1464 | Richmond |
| Full Spectrum Emergency Room At Hardy Oak | 23511 Hardy Oak Blvd | San Antonio |
| Full Spectrum Emergency Room At The Rim | 18007 Ih 10 W | San Antonio |
| Georgetown Family Emergency Center | 1210 W University Ave | Georgetown |
| Golden Triangle Emergency Center | 8035 Memorial Blvd | Port Arthur |
| Golden Triangle Emergency Center | 3107 Edgar Brown Dr | West Orange |
| Grace Er | 1851 Pearland Pkwy | Pearland |
| Grace Er | 10900 Gulf Freeway #B102 | Houston |
| Highland Park Emergency Room | 5150 Lemmon Ave Ste 108 | Dallas |
| Hope Er | 2111 East Denman Avenue | Lufkin |
| Hospitality Health Er | 4222 Seawall Blvd | Galveston |
| Hospitality Health Er | 3111 Mccann Road | Longview |
| Hospitality Health Er | 3943 Old Jacksonville Highway | Tyler |
| Houston Medical Er | 2306 Rayford Rd | Spring |
| Houston Medical Er | 837 Cypress Creek Pkwy Suite 111 | Houston |
| Icare Emergency Center | 1325 S Fm 741 | Forney |
| Icare Emergency Room | 2955 Eldorado Parkway Suite 100 | Frisco |
| Icare Emergency Room | 5500 Sycamore School Rd Suite 150 | Fort Worth |
| Kingwood Er | 2158 Northpark Dr | Kingwood |
| Lakewood Emergency Room | 6101 E Mockingbird Ln | Dallas |
| Laredo Emergency Room | 7510 Mcpherson Rd Ste 101 | Laredo |
| Legacy Er | 8950 N Tarrant Pkwy | North Richland Hills |
| Legacy Er | 9205 Legacy Drive | Frisco |
| Legacy Er | 330 Denton Tap Rd | Coppell |
| Legacy Er | 2810 South Hardin Blvd Suite 100 | Mckinney |
| Legacy Er | 16151 Eldorado Pkwy | Frisco |
| Legacy Er | 1310 West Exchange Parkway | Allen |
| Life Savers Emergency Room | 3820 North Shepherd Drive Suite A | Houston |
| Life Savers Emergency Room | 12665 West Lake Houston Pkwy | Houston |
| Life Savers Emergency Room | 17685 Tomball Parkway | Houston |
| Lonestar 24 Hr Er | 1751 Medical Way | New Braunfels |
| Lubbock Texas Emergency Center Llc | 4337 50Th St | Lubbock |
| Mcallen Emergency Room | 6700 N 10Th Street | Mcallen |
| Medco Er Plano Llc | 3960 Legacy Drive | Plano |
| Memorial Heights Emergency Center | 4000 Washington Avenue, Suite 100 | Houston |
| Memorial Springs Er Llc | 5037 B Fm 2920 | Spring |
| Memorial Village Emergency Room | 14520 Memorial Drive Suite 4 | Houston |
| Mercy Emergency Room | 222 Hwy 6 Suite 100 | Sugar Land |
| My Emergency Room | 2810 South Interstate 35 | San Marcos |
| My Emergency Room 24/7 | 4438 South Clack Street Ste 100 | Abilene |
| Neighbors Emergency Center | 22678 Hwy 59 | Porter |
| Neighbors Emergency Center | 7215 Fairmont Pkwy | Pasadena |
| Neighbors Emergency Center | 14120 Fm 2100 | Crosby |
| Physicians Premier | 12314 Potranco Road Suite 102 | San Antonio |
| Physicians Premier | 580 Cibolo Valley Dr Suite 137 | Cibolo |
| Physicians Premier | 2411 Boonville Road | Bryan |
| Physicians Premier | 11158 Leopard St Ste 103 | Corpus Christi |
| Physicians Premier Emergency Room | 4141 South Staples Suite 106 | Corpus Christi |
| Physicians Premier Emergency Room | 5521 Saratoga Blvd Suite 100 | Corpus Christi |
| Physicians Premier Emergency Room | 7750 South Padre Island Drive | Corpus Christi |
| Physicians Premier Emergency Room | 1860 Highway 181 Suite C | Portland |
| Post Oak Er | 5018 A San Felipe St | Houston |
| Premier Er & Urgent Care | 9110 Jordan Lane Suite 100 | Woodway |
| Premier Er & Urgent Care | 7010 W Adams Ave Suite 100 | Temple |
| Premier Er & Urgent Care | 221 S Jack Kultgen Expy Suite 100 | Waco |
| Premier Er & Urgent Care | 1509 N Interstate 35 Suite 100 | San Marcos |
| Prestige Emergency Room | 738 W Loop 1604 N | San Antonio |
| Prestige Emergency Room | 11590 Galm Rd Ste 110 | San Antonio |
| Prestige Emergency Room | 15140 Nacogdoches Road | San Antonio |
| Prestige Emergency Room Llc | 2810 N Loop 1604 W Suite 110 | San Antonio |
| Prestige Er | 7940 Custer Rd | Plano |
| Prestige Er | 1080 E Cartwright Rd | Mesquite |
| Preston Hollow Emergency Room | 8007 Walnut Hill Lane | Dallas |
| Primecare Emergency Center | 5912 S Cooper St Suite 110 | Arlington |
| Priority Emergency Room | 3800 E 42Nd Street Suite 105 | Odessa |
| Quality Care Er | 2675 41St Street Se Mob #4 Suite 101 | Paris |
| Quality Care Er | 8090 Monty Stratton Parkway | Greenville |
| Rapidcare Emergency Room | 18057 Tx - 105 Suite 220 | Montgomery |
| Rapidcare Emergency Room | 24003 Southwest Fwy | Rosenberg |
| Rapidcare Emergency Room | 1220 W Fairmont Parkway | La Porte |
| Rapidcare Emergency Room | 4885 Hwy 6 | Missouri City |
| Rapidcare Emergency Room | 1510 S Mason Rd | Katy |
| Reliant Er | 3910 Saratoga Blvd | Corpus Christi |
| Rice Emergency Room Llc | 2500 Rice Blvd | Houston |
| Riverside Er Llc | 1860 S Seguin Ave Suite 400 | New Braunfels |
| Sacred Heart Emergency Center | 9774 Katy Freeway, Suite 500 | Houston |
| Schertz Cibolo Emergency Clinic | 4825 Fm 3009 Ste 200 | Schertz |
| Signaturecare Emergency Center - College Station | 1512 S Texas Ave Suite 500 | College Station |
| Signaturecare Emergency Center - Killeen | 800 W Central Texas Expressway | Killeen |
| Signaturecare Emergency Center - Lewisville | 1596 W Main St | Lewisville |
| Signaturecare Emergency Center - Midland | 5409 West Wadley Ave | Midland |
| Signaturecare Emergency Center - Odessa | 2731 N Grandview Ave | Odessa |
| Signaturecare Emergency Center - Pflugerville | 21315 N Sh 130 Bldg 4 | Pflugerville |
| Signaturecare Emergency Center - South Austin | 5701 W Slaughter Ln Bldg G | Austin |
| Signaturecare Emergency Center - Texarkana | 2001 Mall Dr | Texarkana |
| Silverlake Er | 2752 Sunrise Blvd | Pearland |
| South Shore Er Llc | 3016 Marina Bay Dr | League City |
| Spring Emergency Room | 3515 Rayford Road Ste 150 | Spring |
| Star Er | 7007 Indiana Ave | Lubbock |
| Supreme Care Er Lp | 9530 Jones Road | Houston |
| Surepoint Emergency Center Addison | 15240 Dallas Pkwy | Dallas |
| Surepoint Emergency Center Arlington | 4747 Little Road | Arlington |
| Surepoint Emergency Center Azle | 611 Northwest Parkway | Azle |
| Surepoint Emergency Center Denton | 2426 Lillian Miller Parkway | Denton |
| Surepoint Emergency Center Grand Prairie | 901 W Jefferson Street | Grand Prairie |
| Surepoint Emergency Center Hulen | 5900 South Hulen Street | Fort Worth |
| Surepoint Emergency Center Mesquite | 3400 Gus Thomasson Rd | Mesquite |
| Surepoint Emergency Center North Fort Worth Beach | 4551 Western Center Blvd | Fort Worth |
| Surepoint Emergency Center Padre Island | 14433 South Padre Island Drive | Corpus Christi |
| Surepoint Emergency Center Richardson | 15767 North Coit Road | Dallas |
| Surepoint Emergency Center Rowlett | 3301 Lakeview Pkwy | Rowlett |
| Surepoint Emergency Center Stephenville | 2108 W Washington Street | Stephenville |
| Surepoint Emergency Center Weatherford | 730 Adams Drive | Weatherford |
| Swiftstar Emergency | 24433 Katy Fwy #700 | Katy |
| Swiftstar Emergency | 2320 South Shepherd | Houston |
| Texas Emergency Care Center | 3115 Dixie Farm Rd #107 | Pearland |
| The Emergency Center At Alamo Ranch Llc | 11320 Alamo Ranch Parkway | San Antonio |
| The Emergency Clinic At Alamo Heights | 6496 N New Braunfels Ave | San Antonio |
| The Emergency Clinic At The Pearl | 2015 Broadway St Suite B | San Antonio |
| The Emergency Room At Katy Main Street | 25765 Katy Freeway | Katy |
| Top Care Er Llc | 1324 N Shepherd Dr Ste 100 | Houston |
| Total Care | 800 W Belt Line Rd | Desoto |
| Total Care | 1101 University Drive | Fort Worth |
| Total Care | 850 N Highway 67 | Cedar Hill |
| Total Care | 3321 S Cooper St | Arlington |
| Total Care | 709 South Main Street | Weatherford |
| Total Care | 5245 Preston Road | Frisco |
| Total Care | 738 Centerville Road | Garland |
| Total Care Denton | 3111 Teasley Lane | Denton |
| Totalcare Emergency | 8501 Benbrook Blvd Suite 103 | Benbrook |
| Total Point Emergency Center | 300 Se John Jones Dr | Burleson |
| Total Point Emergency Center | 8200 N Macarthur Blvd 110 | Irving |
| Total Point Emergency Center | 5000 West Eldorado Parkway | Mckinney |
| Total Point Emergency Center | 501 N Brentwood Dr | Lufkin |
| Total Point Emergency Center | 8929 Spring Cypress Rd | Spring |
| Total Point Emergency Center | 10705 E Northwest Hwy | Dallas |
| Total Point Emergency Center - Conroe | 3840 W Davis St | Conroe |
| Total Point Emergency Center - Cypress | 20440 West Rd | Cypress |
| Total Point Er Missouri City | 8927 Hwy 6 | Missouri City |
| Trinity Er | 3022 Trawood #A | El Paso |
| Victoria Er | 6703 N Navarro | Victoria |

**Table S3.** Parameters Mandated by Texas HB 2041 (2019)

| **Category** | **Parameter** | **TX Statute Section** | **Bill Text** | **Operational Definition** | **Related Satellite FrED Rules** |
| --- | --- | --- | --- | --- | --- |
| General facility identification | States that Facility is a FrED | Sec. 254.155(a)(1)(A) | "the facility is a freestanding emergency medical care facility;" | Compliant if the facility refers to itself using any variation of “freestanding emergency” followed by terms like department, facility, or medical care facility. | 241.252 (b)(1) (B) |
| Facility fee | States that Facility Charges Facility Fee | Sec. 254.155(a)(1)(B) | "the facility charges rates comparable to a hospital emergency room and may charge a facility fee;" | Compliant if the disclosure includes language indicating the facility may charge a facility fee. | 241.252 (b)(1) (B) |
|  | Mentions Comparable Charges to Hospital ER | Sec. 254.155(a)(1)(B) | "the facility charges rates comparable to a hospital emergency room and may charge a facility fee;" | Compliant if the facility states that its rates are comparable to those of a hospital emergency room. | 241.252 (b)(1) (B) |
|  | States the Median Facility Fee | Sec. 254.156(d)(3)(A)(i) | "(A) state 'This facility charges a facility fee for medical treatment' and include: (i) the facility’s median facility fee;" | Compliant if the facility explicitly states the median facility fee; the patient should not need to calculate it themselves. | 241.205 |
|  | States the Range of Facility Fees | Sec. 254.156(d)(3)(A)(ii) | "(A) state 'This facility charges a facility fee for medical treatment' and include: (ii) a range of possible facility fees;" | Compliant if the facility provides a clear range of potential facility fees. A chargemaster list satisfies this requirement. | 241.205 |
|  | States the Facility Fee at Each Level | Sec. 254.156(d)(3)(A)(iii) | "(A) state 'This facility charges a facility fee for medical treatment' and include: (iii) the facility fees for each level of care provided at the facility;" | Compliant if the fees are broken down by level of care. A chargemaster list is acceptable. | 241.205 |
| Observation fee | States that Facility Charges Observation Fee | Sec. 254.156(d)(3)(B) | "(B) state 'This facility charges an observation fee for medical treatment' and include:" | Compliant if the disclosure indicates the facility may charge an observation fee. | 241.205 |
|  | States the Median Observation Fee | Sec. 254.156(d)(3)(B)(i) | "(B) state 'This facility charges an observation fee for medical treatment' and include: (i) the facility’s median observation fee;" | Compliant if the median observation fee is explicitly stated, without requiring patient calculation. | 241.205 |
|  | States the Range of Observation Fees | Sec. 254.156(d)(3)(B)(ii) | "(B) state 'This facility charges an observation fee for medical treatment' and include: (ii) a range of possible observation fees;" | Compliant if a clear range of observation fees is provided. A chargemaster list fulfills this requirement. | 241.205 |
|  | States the Observation Fee at Each Level | Sec. 254.156(d)(3)(B)(iii) | "(B) state 'This facility charges an observation fee for medical treatment' and include: (iii) the observation fees for each level of care provided at the facility." | Compliant if observation fees are listed by level of care. A chargemaster list is acceptable. | 241.205 |
| Insurance information | Clear "Insurance Information" Section | Sec. 254.155(b)(4)(A-B), Sec. 254.155(2) | "...on the home page of the facility’s Internet website or on a different page available through a hyperlink that is: (A) entitled 'Insurance Information'; and (B) located prominently on the home page." | Compliant if the website includes a section with “Insurance” in the title (e.g., “Insurance,” “Insurance Section,” or “Insurance and Transparency”) and contains the required information, even if it is not on a separate page. | 251.252.(b)(2)(B) |
|  | Mentions Out-of-Network Provider for Benefit Plan | Sec. 254.155(a)(1)(C) | "a facility or a physician providing medical care at the facility may be an out-of-network provider for the patient’s health benefit plan provider network;" | Compliant if the facility or physician discloses that they may be out-of-network for the patient’s insurance plan, unless they are actually in-network. | 241.252(b)(1)(c) |
|  | Does NOT Claim to be In-Network unless officially a network provider for the benefit plan | Sec. 254.157(a) | "A facility may not advertise or hold itself out as a network provider of an insurer, health maintenance organization, or health benefit plan network, including by stating that the facility 'takes' or 'accepts' any insurer, health maintenance organization, health benefit plan, or health benefit plan network, unless the facility is a network provider of a health benefit plan issuer." | Compliant if the facility does not present itself as in-network for any insurer or plan unless it is formally part of that network. | 241.202 (2) |
|  | Does NOT use "takes" or "accepts" for insurers, HMOs, or benefit plans unless in-network | Sec. 254.157(a) | "A facility may not advertise or hold itself out... including by stating that the facility 'takes' or 'accepts' any insurer, health maintenance organization, health benefit plan, or health benefit plan network, unless the facility is a network provider..." | Compliant if the facility avoids using terms like “takes” or “accepts” when referring to insurance plans it is not in-network with. | 241.202 (2) |
|  | Does NOT display name or logo of benefit plan if out-of-network | Sec. 254.157(b) | "A facility may not post the name or logo of a health benefit plan issuer in any signage or marketing materials if the facility is an out-of-network provider for all of the issuer’s health benefit plans." | Compliant if the facility does not show the name or logo of any health plan for which it is not an in-network provider. | 241.202 (2) |
|  | Mentions Physician May Bill Separate From Facility | Sec. 254.155(a)(1)(D) | "a physician providing medical care at the facility may bill separately from the facility for the medical care provided to a patient;" | Compliant if it is clearly stated that physicians providing care may bill separately from the facility. | 241.252(b)(1)(D) |
| **Notes**: All independent and satellite FrEDs must comply with HB2041 guidelines. Referenced hospital-based FrED requirements are listed separately, as applicable. | | | | | |

**Table S4.** Characteristics of Cities based on FrED Location, 2024

|  | **Independent FrEDs** | **Hospital FrEDs** | **P-Value** |
| --- | --- | --- | --- |
| N | 207 (59.0%) | 144 (41.0%) |  |
| Population Total | 557,185 (742,852) | 605,974 (856,805) | 0.57 |
| Median HH Income | $42,848 ($13,061) | $39,224 ($11,107) | 0.007 |
| % Commercial Insurance | 66.67 (12.76) | 62.56 (14.25) | 0.005 |

Source: List of facilities required to report emergency department data obtained from the Texas Department of State Health Services (DSHS), Texas Health Care Information Collection (THCIC). Population estimates by place were obtained from the 2024 ACS 5-year estimates. Notes: FrEDs are owned by a hospital. Independent FrEDs are independently owned/operated. The percentage of the population with commercial insurance represents the share of the nonelderly population (0-64 years) with commercial (employer sponsored or direct purchase) health insurance coverage. Standard deviations are provided in parentheses. FrED facilities located in Cypress, Kingwood, and Porter neighborhoods were classified as Houston to conduct analysis by city.

**Table S5.** Distribution of Texas FrEDs by City

| **City** | **Number of Ind. FrEDs** | **Average Compliance Rate** | **City** | **Number of Satellite FrEDs** | **Average Compliance Rate** |
| --- | --- | --- | --- | --- | --- |
| Houston | 15 | 70.83% | Houston | 17 | 51.84% |
| San Antonio | 14 | 66.52% | San Antonio | 12 | 45.83% |
| Dallas | 11 | 60.80% | El Paso | 8 | 42.19% |
| Austin | 9 | 69.44% | Laredo | 5 | 35.00% |
| Fort Worth | 8 | 64.84% | Amarillo | 4 | 62.50% |
| Corpus Christi | 7 | 59.82% | Spring | 4 | 34.38% |
| Frisco | 6 | 77.08% | Cypress | 3 | 52.08% |
| Spring | 5 | 63.75% | Edinburg | 3 | 41.67% |
| Amarillo | 3 | 75.00% | Pearland | 3 | 54.17% |
| Arlington | 3 | 72.92% | Baytown | 2 | 65.63% |
| Cypress | 3 | 62.50% | Fort Worth | 2 | 62.50% |
| Katy | 3 | 52.08% | Humble | 2 | 62.50% |
| Mesquite | 3 | 66.67% | Katy | 2 | 28.13% |
| Odessa | 3 | 70.83% | Kingwood | 2 | 46.88% |
| Pearland | 3 | 52.08% | League City | 2 | 28.13% |
| Tyler | 3 | 56.25% | Leander | 2 | 43.75% |

Source: List of facilities required to report emergency department data obtained from the Texas Department of State Health Services (DSHS), Texas Health Care Information Collection (THCIC). Notes: The data were obtained on November 11, 2024. Satellite FrEDs are owned by a hospital. Independent FrEDs are independently owned/operated.

**Table S6.** Texas HB 2041 Compliance by Website Sharing and Facility Type

|  | **Number of FrEDs** | **Average Compliance Rate** | **P-Value** |
| --- | --- | --- | --- |
| Not Sharing a Website: |  |  |  |
| Hospital FrED | 15 | 42.92% | <0.001 |
| Independent FrED | 55 | 65.34% |  |
| Sharing a Website: |  |  |  |
| Hospital FrED | 129 | 45.16% | <0.001 |
| Independent FrED | 152 | 66.33% |  |

Source: List of facilities required to report emergency department data obtained from the Texas Department of State Health Services (DSHS), Texas Health Care Information Collection (THCIC). Notes: The data were obtained on November 11, 2024. Satellite FrEDs are owned by a hospital. Independent FrEDs are independently owned/operated. Facilities were classified as sharing a website if their information was presented at the same web address with distinct links for each location.
